# Short-term prognostic value of forced expiratory volume in 1 second divided by height cubed in a prospective cohort of people 80 years and older

**DOI:** 10.1186/s12877-015-0013-4

**Published:** 2015-02-25

**Authors:** Eralda Turkeshi, Bert Vaes, Elena Andreeva, Catharina Matheï, Wim Adriaensen, Gijs Van Pottelbergh, Jean-Marie Degryse

**Affiliations:** Institute of Health and Society, Université Catholique de Louvain (UCL), Clos Chapelle-aux-Champs 30, bte B1.30.15, 1200, Brussels, Belgium; Department of Public Health and Primary Care, Katholieke Universiteit Leuven (KUL), Kapucijnenvoer 33, blok J, PB 7001 3000, Leuven, Belgium

## Abstract

**Background:**

Spirometry-based parameters of pulmonary function such as forced expiratory volume in 1 second (FEV_1_) have prognostic value beyond respiratory morbidity and mortality. FEV_1_ divided by height cubed (FEV_1_/Ht^3^) has been found to be better at predicting all-cause mortality than the usual standardization as percentage of predicted "normal values" (FEV_1_%) and its use is independent of reference equations. Yet, limited data are available on the very old adults (80 years and older) and in association to other adverse health outcomes relevant for this age group. This study aims to investigate the short-term prognostic value of FEV_1_/Ht^3^ for all-cause mortality, hospitalization, physical and mental decline in a cohort of very old adults.

**Methods:**

In a population-based prospective cohort study of 501 very old adults in Belgium, comprehensive geriatric assessment and spirometry were performed at baseline and after 1.7 ± 0.21 years. Kaplan-Meier curves for 3-year all-cause mortality and hospitalization rates and multivariable analysis adjusted for age, sex, smoking status, co-morbidities, anemia, high C reactive protein and creatinine levels examined the association of FEV_1_/Ht^3^ with all-cause mortality, unplanned hospitalization and decline in mental and physical functioning. Physical functioning was assessed by activities of daily living, a battery of physical performance tests and grip strength. Mental functioning was assessed with mini mental state examination and 15 items geriatric depression scale.

**Results:**

Individuals in the lowest quartile of FEV_1_/Ht^3^ had a statistically significant increased adjusted risk for all-cause mortality (hazard ratio [HR] 1.69, 95% confidence interval [CI] 1.10-2.60) and unplanned hospitalization (HR 1.65, 95% CI 1.21-2.25), as well as decline in physical (odds ratio [OR] 1.89, 95% CI 1.05-3.39) and mental functioning (OR 2.39, 95% CI 1.30-4.40) compared to the rest of the study population.

**Conclusions:**

In a cohort of very old adults, low FEV_1_ expressed as FEV_1_/Ht^3^ was found to be a short-term predictor of all-cause mortality, hospitalization and decline in physical and mental functioning independently of age, smoking status, chronic lung disease and other co-morbidities. Further research is needed on FEV_1_/Ht^3^ as a potential risk marker for frailty and adverse health outcomes in this age group.

## Background

The prognostic value of the spirometry parameters of pulmonary function (PF) such as forced expiratory volume in 1 second (FEV_1_) and forced vital capacity (FVC) has been found to extend beyond respiratory morbidity and mortality into overall morbidity, mortality and other adverse health outcomes [1-5]. Inflammatory markers seem to have a significant role in the multiple pathways that link PF to overall mortality and morbidity [4,6]. Impaired PF has also been found to be associated with reduced physical performance and disability in community-dwelling adults [7-10], while lower PF measures at midlife have been found to be independently associated with lower cognitive performance in later life [7,11-17]. Recently, cross-sectional and longitudinal analysis has also shown that impaired PF and frailty are strongly associated and both increase the risk for mortality in older adults [18], leading to further interest in researching the role of PF parameters as prognostic markers of adverse health outcomes in this worldwide growing age group.

Yet the interpretation of key spirometry parameters is an area of ongoing discussion, especially for the older adults [19]. The common approach of expressing FEV_1_ as percentage of a predicted “normal” value (FEV_1_%) is dependent on reliable age-specific reference values derived from spirometry data of "healthy" people of equivalent age and gender, but these have been relatively lacking for the very old adults until recently when the Global Lung Initiative (GLI) all-age reference equations have been made available for populations up to 95 years old [20,21]. Yet, the GLI reference equations need to be validated for people over 80 years old and more data are needed for this age group [21]. The FEV_1_% approach also does not account for the variability of predicted values that is even higher in the older adults [20,22]. These limitations of FEV_1_% have lead to exploration of alternative ways of standardizing FEV_1_ such as FEV_1_ standardized residuals (FEV_1_SR or z-scores), FEV_1_ divided by height squared or cubed (FEV_1_/Ht^3^) or as a function of the sex-specific first percentile (FEV1 quotient (FEV_1_Q) with the last two being found to be superior to FEV_1_% in predicting all-cause mortality [23-27]. Studies on the ability of these alternative expressions of FEV_1_ to predict mortality in the very old adults and other relevant adverse health outcomes for this age group are very limited.

The aim of this study is to investigate the short-term prognostic value of FEV_1_/Ht^3^ for all-cause mortality and unplanned hospitalizations as well as declines in physical and mental functioning in a cohort of very old adults. FEV_1_/Ht^3^ was investigated in this study as its use is independent of reference equations.

## Methods

### Study design and population

**Figure 1:**
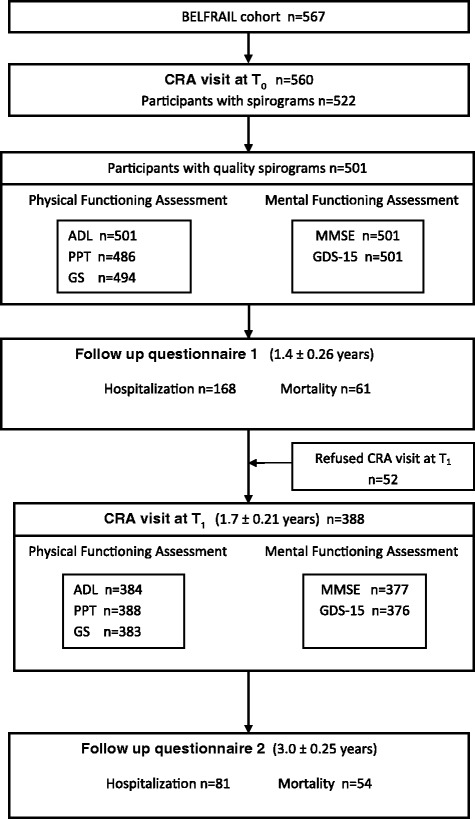
**Flowchart of the data collection in the BELFRAIL study CRA: clinical research assistant; GP: general practitioner; ADL: activities of daily living; PPT: physical performance tests; GS: grip strength; MMSE: mini mental state examination; GDS-15: 15 items geriatric depression scale.**

The BELFRAIL study (BF_c80+_) is a prospective, observational, population-based cohort study of people aged 80 years or older living in Belgium aiming to acquire a better understanding of the epidemiology and pathophysiology of chronic diseases in this age-group and to study the dynamic interaction between health, frailty and disability in a multi-system approach. The study protocol and sampling methods have been already described [28]. Briefly, between November 2008 and September 2009, in 29 general practice centers, 567 individuals aged 80 years and older were included in the BF_c80+_, excluding only those with severe dementia (defined as a mini-mental state examination ≤15/30) and those in palliative or emergency care. At baseline (T_0_), the participants' general practitioners (GP) recorded socio-demographic data and medical history. An extensive assessment by a clinical research assistant (CRA) included performance tests, questionnaires and technical examinations such as spirometry as well as collection of blood samples for laboratory tests. The same comprehensive assessment was repeated at 1.7 ± 0.21 years from baseline (T_1_). Hospitalization and mortality data were collected periodically until 3.0 ± 0.25 years from baseline (Figure 1). The study protocol was approved by the Biomedical Ethics Committee of the Medical School of the Universite Catholique de Louvain (UCL) in Brussels, Belgium. All participants gave written informed consent.

### Baseline spirometry

All spirometry data were gathered by two trained CRAs using a Spirobank spirometer (Medical International Research, Rome, Italy) that has been previously found to be reliable for research purposes [29]. After demonstration of the correct spirometry maneuver, participants performed up to eight forced vital capacity maneuvers or until exhaustion to achieve the acceptability and repeatability criteria of the American Thoracic Society (ATS)/European Respiratory Society (ERS) [30]. Repeatability of the spirometry was calculated automatically in accordance with these criteria. Two independent researchers evaluated all spirograms by the ATS/ERS criteria and classified them in the following levels: 1 – all criteria concerning acceptability and repeatability are fulfilled; 2 – all criteria are fulfilled except duration of expiration > 6 seconds; 3 –spirograms have good starts and no cough during the 1st second of manoeuvre; 4 – none of the criteria are fulfilled. Standardized measurements of height were performed during the CRA visit at T_0_. FEV_1_ was expressed as FEV_1_ divided by height cubed and ranked in quartiles.

### All-cause mortality and hospitalizations

The detailed follow-up questionnaires received from the GPs of the participants at 1.4 ± 0.26 years and 3.0 ± 0.25 years after the baseline (Figure 1) reported on the date and cause of mortality and hospitalizations. All-cause mortality and time to first unplanned hospitalization after the CRA visit at T_0_ were used as outcome measurements.

### Physical decline

Activities of daily living (ADL), physical performance tests (PPT) and grip strength were used as measures of physical functioning. Physical decline was defined as a relevant decline in any of these tests between T_0_ and T_1_.

The ADL consisted of asking the respondents to describe the degree of difficulty they have with six activities of daily living: climbing stairs, walking 5 minutes outdoors without resting, getting up and sitting down in a chair, dressing and undressing oneself, using own or public transport and cutting one's own nails [31]. The response categories ranged from 1 (“No, I cannot”) to 5 (“Yes, without difficulty") with the total score of 6–30. The relevant decline was determined using the Edwards-Nunnally index that determines the probability of substantial individual change and avoids the problem of regression to the mean [32]. This index computes whether a significant change has occurred between T_0_ and T_1_ based on the reliability of the scale and the 95% Confidence Interval (CI) of the score at T_0_.

The PPT consisted of measured times of walking 3 meters and return, sitting and standing from a chair, putting on and taking off a cardigan and maintaining balance in a tandem stand [33-35]. For the first three tasks, those who could not complete the task were assigned a score of 0, while those completing it were scored 1–4 depending on the quartile of time needed to complete the task (fastest time scored as 4). For the balance in tandem stand, those who could not perform it or maintained the balance for less than 3 seconds were assigned a score of 0, while those maintaining it until ≤9 seconds or more were scored 1 and 2 respectively. The summary performance score (range 0–14) was calculated by adding up the category scores and ranked into gender-specific quintiles. The relevant decline was defined as a drop by at least two gender-specific quintiles in total score between T_0_ and T_1_.

The grip strength was measured in the dominant hand using a JAMAR® Plus digital handheld dynamometer [36,37]. Three attempts at maximal squeeze were recorded. The relevant decline was defined as a drop by at least two gender-specific quintiles of the best attempt between T_0_ and T_1_.

### Mental decline

The mini-mental state examination (MMSE) and the 15-item Geriatric Depression Scale (GDS-15) were used to assess the cognitive and mood/affective components of the mental status respectively. Mental decline was defined as a relevant decline in any of these tests between T_0_ and T_1_.

The MMSE evaluates cognitive efficiency by examining orientation in time and space; short and middle term memory; calculation; comprehension and constructive praxis with scores range from 0 to 30 points (optimal) [38]. The relevant decline was determined using the Edwards-Nunnally index [32].

The GDS-15 has been designed to screen for depression in the older adults and has been found to have good accuracy in the very old adults [39,40]. Its scores range from 0 (optimal) to 15. The relevant decline was determined as a shift of score from <5 to ≥5 [41].

### Potential confounding variables

In addition to age and sex, other potential confounding variables associated with PF and the study outcomes [4,6,16] were included in the statistical analysis as follows: smoking status (never, previous or current smoker), high sensitivity C-Reactive Protein (hs-CRP) > 0.3 mg/dl, serum creatinine > 2 mg/dL, anemia (hemoglobin < 12 g/dL for women and <13 g/dL for men), history of cardiovascular disease (CVD), osteoporosis, Parkinson disease, diabetes, hypertension as well as chronic respiratory morbidities such as chronic obstructive pulmonary disease (COPD) and asthma. The GP reported the recorded status of smoking and the presence of morbidities at T_0_. Plasma (EDTA) and serum samples were stored and frozen in −8°C until analysis. Creatinine and hs-CRP were measured in serum using the UniCel DxC 800 Synchron (Beckman-Coulter, Brea, USA). Hemoglobin was measured on whole blood using the Sysmex XE-2100 automated hematology analyzer (Milton Keynes, UK).

### Statistical analysis

Descriptive statistics were calculated for baseline and outcome variables. Continuous variables are presented as mean ± standard deviation (SD) or median and inter-quartile range (IQR), while categorical ones are presented as numbers and frequencies. FEV_1_/Ht^3^ values were ranked into two groups based on the quartiles of their distribution: those in the lowest quartile and the rest of the study population. Comparison of baseline and outcome variables across the FEV_1_/Ht^3^ quartiles was tested using one way analysis of variance for parametric variables, Kruskall-Wallis test for non-parametric variables and Pearson's Chi-Square test for categorical variables. Kaplan-Meier curves for all-cause mortality and hospitalization during 3 years follow-up for the lowest FEV_1_/Ht^3^ quartile and the rest of the population were compared using a log-rank test. Cox proportional hazards regression model was used to estimate the hazard ratio (HR) for mortality and hospitalization for the lowest FEV_1_/Ht^3^ quartile adjusting for potential confounders in three consecutive models. Logistic regression model (adjusted for the abovementioned confounders in three consecutive models) was used to estimate the odds ratios (OR) of the lowest FEV_1_/Ht^3^ quartile for decline in physical and mental functioning. The rest of the study population was used as reference category. Variables were first checked for multicollinearity. A two-tailed probability value p < 0.05 was considered statistically significant. Statistical analysis was performed using SPSS 22.0 for Windows (SPSS Inc., Chicago, IL, USA).

## Results

### Baseline characteristics of the study population

The BF_c80+_ cohort consisted of 567 participants and 522 of them performed spirometry during the CRA visit at T_0_. The quality of spirograms was scored as ATS 1 in 226 participants (43.3%), ATS 2 in 214 (41%), ATS 3 in 61 (11.7%) and ATS 4 in 21 (4%). Participants with spirograms scored as ATS 1–3 (501) were included in the study population for our analysis (Figure 1). They were comparable to the initial BF_c80+_ population with a mean age of 84.79 (SD 3.66) and 186 (37.1%) men. The main baseline demographic and clinical characteristics of the study population in total and by FEV_1_/Ht^3^ quartiles are shown in Table 1. The differences in age and gender across the FEV_1_/Ht^3^ quartiles were statistically significant with more female and older participants in the lowest FEV_1_/Ht^3^ quartile. Current or previous smoking was present in 31.6% of the population with no statistically significant differences in smoking status between the FEV_1_/Ht^3^ quartiles. All of the components of physical and mental functioning at baseline (ADL, PPT, grip strength, MMSE and GDS-15) showed a statistically significant difference across the FEV_1_/Ht^3^ quartiles with worse scores in the lowest FEV_1_/Ht^3^ quartile. The prevalence of asthma and COPD in the study population was 4.8% and 10.8% respectively. The differences in prevalence of asthma, COPD and osteoporosis across the FEV_1_/Ht^3^ quartiles were statistically significant with higher prevalence in the lowest quartile.

**Table 1:** **Baseline characteristics of the study population in total and across the FEV**_**1**_ **/Ht** ^**3**^ **quartiles**

	**Total population (n = 501)**	**Lowest quartile (n = 129)**	**Second quartile (n = 111)**	**Third quartile (n = 135)**	**Highest quartile (n = 126)**	**p value**
Age (years), mean ± SD	84.8 ± 3.7	85.6 ± 3.8	85.3 ± 3.8	84.7 ± 3.7	83.6 ± 3.0	0.00^a^
Male, n (%)	186 (37.1)	30 (23.3)	34 (29.6)	51 (38.1)	69 (54.8)	0.00^b^
BMI (kg/m^2^), mean ± SD	27.4 ± 4.9	26.7 ± 5.4	27.7 ± 4.9	28.1 ± 5.3	27.0 ± 3.7	0.11^a^
FEV_1_/Ht^3^, mean ± SD	0.4 ± 0.1	0.3 ± 0.1	0.4 ± 0.0	0.4 ± 0.0	0.6 ± 0.1	0.00^a^
FEV_1_, mean ± SD	1.7 ± 0.6	1.1 ± 0.3	1.5 ± 0.3	1.8 ± 0.3	2.4 ± 0.5	0.00^a^
Smoker/ex-smoker, n (%)	158 (31.6)	41 (32)	34 (29.6)	35 (26.1)	46 (36.5)	0.24^b^
COPD, n (%)	54 (10.8)	30 (23.4)	11 (9.6)	4 [17]	8 (6.3)	0.00^b^
Asthma, n (%)	24 (4.8)	12 (9.4)	5 (4.3)	5 (3.7)	2 (1.6)	0.03^b^
CVD history, n (%)	256 (51.1)	77 (60.2)	69 (60)	71 (53)	58 (46)	0.08^b^
Hypertension, n (%)	356 (71.2)	97 (75.8)	87 (75.7)	86 (64.2)	87 (69.1)	0.13^b^
Diabetes, n (%)	95 (19)	23 (18)	25 (21.7)	31 (23.1)	16 (12.7)	0.22^b^
Parkinson, n (%)	9 (1.8)	2 (1.6)	0 (0)	4 [17]	3 (2.4)	0.34^b^
Osteoporosis, n (%)	107 (21.7)	40 (31.7)	32 (28.6)	23 (17.3)	15 (12.1)	0.00^b^
Anemia, n (%)	99 (20)	25 (19.7)	25 (21.7)	23 (17.4)	27 (21.8)	0.90^b^
hs-CRP (mg/dl), median [IQR]	0.18 [0.1-0.4]	0.22 [0.1-0.6]	0.17 [0.1-0.4]	0.15 [0.1-0.3]	0.15 [0.1-0.4]	0.02^c^
Serum creatinine (mg/dl), median [IQR]	0.95 [0.8-1.2]	0.92 [0.7-1.2]	0.93 [0.8-1.1]	0.90 [0.8-1.2]	1.0 [0.8-1.2]	0.17^c^
ADL score at T_0_, median [IQR]	25 [21–27]	22 [17.3-26]	25 [21–27]	25 [22–28]	27 [24–29]	0.00^c^
PPT score at T_0_, median [IQR]	9 [5–11]	7 [4–9]	8 [5–11]	8 [6–11]	10 [7–12]	0.00^c^
Grip strength at T_0_, median [IQR]	20 [15.1-26.3]	16.9 [12.7-21.3]	18.7 [14.8-23]	20.2 [15.5-26.2]	25.3 [19.4-33.9]	0.00^c^
MMSE score at T_0_, median [IQR]	28 [26–29]	27 [24.3-29]	28 [26–29]	28 [25.3-29]	28 [27–30]	0.00^c^
GDS-15 score at T_0_, median [IQR]	2 [1–4]	2 [2–5]	3 [1–4.3]	2 [1–3]	2 [1–3]	0.00^c^

BMI: body mass index; FEV_1_/Ht^3^: forced expiratory volume in one second over height cubed; COPD: chronic obstructive pulmonary diseases; CVD: cardiovascular disease; hs-CRP: high sensitivity C-reactive protein; ADL: activities of daily living; T_0_: baseline assessment; PPT: physical performance tests; MMSE: mini-mental state examination; GDS: geriatric depression scale; ^a^based on one way ANOVA; ^b^based on Pearson Chi-Square; ^c^based on Kruskall-Wallis.

### All-cause mortality and hospitalization

**Figure 2:**
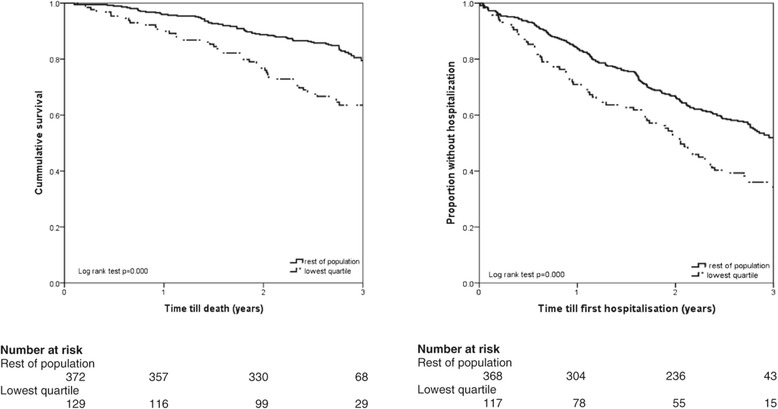
**Kaplan-Meier survival curves for 3 years all-cause mortality and hospitalization for the lowest quartile of FEV**_**1**_ **/Ht** ^**3**^ **and rest of study population.**

Follow-up data for mortality were available for all the participants (501), while data on hospitalizations were available for 494 of them. During the 3.0 ± 0.25 years of follow up, 115 (23%) participants died and 249 (50.4%) had at least one hospitalization reported. Kaplan-Meier survival curves showed significant higher all-cause mortality and hospitalization at 3 years follow up for the participants in the lowest FEV_1_/Ht^3^ quartile (Figure 2). Even after adjustment for the potential confounders in the Cox proportional hazards model, participants with FEV_1_/Ht^3^ in the lowest quartile had a higher risk of all-cause mortality and hospitalizations with adjusted HR of 1.69 (95% CI 1.10-2.60, p < 0.05) and 1.65 (95% CI 1.21-2.25, p < 0.01) respectively (Table 2).

**Table 2:** **Multivariable Cox regression analysis for all-cause mortality and hospitalization**

	**Mortality (N = 478)**				**Hospitalization (N = 463)**			
	**Hazard ratio (95%** **confidence interval)**							
**Variables**	**Unadjusted model**	**Model 1**	**Model 2**	**Model 3**	**Unadjusted model**	**Model 1**	**Model 2**	**Model 3**
Lowest FEV_1_/Ht^3^ quartile	2.13 (1.46-3.09)**	2.13 (1.44-3.14)**	1.77 (1.16-2.70)**	1.69 (1.10-2.60)*	1.68 (1.27-2.21)**	1.73 (1.30-2.31)**	1.56 (1.15-2.11)**	1.65 (1.21-2.25)**
Age		1.08 (1.03-1.13)**	1.07 (1.02-1.12)**	1.06 (1.01-1.11)*		1.04 (1.01-1.08)*	1.04 (1.00-1.07)	1.02 (0.99-1.06)
Male		1.44 (0.98-2.11)	1.08 (0.64-1.81)	0.91 (0.53-1.57)		1.42 (1.09-1.85)*	1.19 (0.82-1.74)	0.99 (0.67-1.47)
Smoker or ex-smoker			1.34 (0.81-2.21)	1.41 (0.86-2.34)			1.40 (0.98-2.00)	1.50 (1.04-2.14)*
History of CVD			1.48 (0.99-2.21)	1.45 (0.96-2.18)			1.46 (1.11-1.91)**	1.37 (1.04-1.82)*
Hypertension			1.61 (1.01-2.55)*	1.53 (0.95-2.48)			1.25 (0.92-1.68)	1.25 (0.92-1.70)
Diabetes			0.72 (0.42-1.21)	0.68 (0.40-1.15)			1.19 (0.86-1.65)	1.09 (0.78-1.52)
Parkinson disease			1.43 (0.35-5.94)	1.76 (0.42-7.35)			0.69 (0.21-2.20)	0.91 (0.28-2.94)
Osteoporosis			1.18 (0.74-1.86)	1.24 (0.77-1.99)			1.51 (1.08-2.09)*	1.55 (1.10-2.17)*
Asthma			1.01 (0.43-2.37)	0.99 (0.42-2.33)			1.50 (0.85-2.65)	1.39 (0.78-2.49)
COPD			1.52 (0.87-2.68)	1.56 (0.87-2.78)			1.08 (0.70-1.65)	1.02 (0.65-1.59)
Anemia				1.53 (0.98-2.38)				1.56 (1.14-2.15)**
hs-CRP > 0.3 mg/dl				1.37 (0.93-2.03)				1.27 (0.96-1.67)
Serum creatinine > 2 mg/dl				1.53 (0.72-3.26)				2.30 (1.32-4.03)**

FEV_1_/Ht^3^: Forced expiratory volume in one second divided by height cubed; CVD: cardiovascular disease; COPD: chronic obstructive pulmonary diseases; hs-CRP: high sensitivity C-Reactive Protein; Model 1: adjusted for age and sex; Model 2: Model 1+ smoking status and other co-morbidities reported by GP; Model 3: Model 2+ anemia, hs-CRP and serum creatinine; *p-value < 0.05 **p-value < 0.01.

### Physical and mental decline

Complete data regarding physical and mental decline after 1.7 ± 0.21 years follow-up were available for 378 participants. As defined for the purposes of this analysis, physical and mental decline were identified respectively in 116 (30.7%) and 85 (22.5%) participants. The association between the lowest quartile of FEV_1_/Ht^3^ and physical decline was not statistically significant in unadjusted analysis. After adjustment for anemia, high CRP and creatinine, a significant positive association was found between the lowest FEV_1_/Ht^3^ quartile and physical decline with OR 1.89 (95%CI 1.05-3.39 p < 0.05). Participants in the lowest FEV_1_/Ht^3^ quartile had a statistically significant increased risk for mental decline with adjusted OR of 2.39 (95% CI 1.30-4.40 p < 0.01) (Table 3).

**Table 3:** **Multivariable logistic regression analysis for physical and mental decline**

	**Physical decline (N = 362)**				**Mental decline (N = 362)**			
	**Odds ratio (95%** **Confidence interval)**							
**Predictor variables**	**Unadjusted model**	**Model 1**	**Model 2**	**Model 3**	**Unadjusted model**	**Model 1**	**Model 2**	**Model 3**
Lowest FEV_1_/Ht^3^ quartile	1.54 (0.93-2.56)	1.45 (0.86-2.46)	1.70 (0.96-3.01)	1.89 (1.05-3.39)*	2.54 (1.49-4.32)**	2.36 (1.37-4.06)**	2.39 (1.32-4.33)**	2.39 (1.30-4.40)**
Age		1.11 (1.04-1.18)**	1.11 (1.04-1.19)**	1.10 (1.03-1.18)**		1.11 (1.04-1.19)**	1.10 (1.02-1.18)**	1.08 (1.00-1.17)*
Male		1.06 (0.66-1.70)	0.94 (0.49-1.79)	0.95 (0.49-1.90)		0.90 (0.53-1.54)	0.71 (0.34-1.48)	0.70 (0.32-1.50)
Smoker or ex-smoker			1.15 (0.60-2.20)	0.96 (0.49-1.90)			1.55 (0.75-3.21)	1.68 (0.79-3.56)
History of CVD			1.13 (0.70-1.83)	1.09 (0.67-1.80)			1.17 (0.68-2.00)	1.18 (0.68-2.06)
Hypertension			1.16 (0.69-1.96)	1.15 (0.66-1.98)			1.44 (0.80-2.62)	1.49 (0.80-2.78)
Diabetes			0.69 (0.37-1.29)	0.63 (0.33-1.19)			0.38 (0.17-0.84)*	0.40 (0.18-0.87)*
Parkinson disease			4.00 (0.56-28.46)	2.38 (0.27-21.45)			3.65 (0.47-28.42)	1.46 (0.10-20.41)
Osteoporosis			0.62 (0.33-1.16)	0.62 (0.33-1.17)			0.93 (0.48-1.82)	0.90 (0.45-1.79)
Asthma			2.41 (0.80-7.24)	2.63 (0.88-7.88)			1.01 (0.25-4.05)	1.09 (0.27-4.40)
COPD			0.44 (0.16-1.20)	0.47 (0.17-1.29)			0.52 (0.18-1.50)	0.50 (0.17-1.47)
Anemia				1.65 (0.89-3.04)				0.94 (0.46-1.91)
hs-CRP > 0.3 mg/dl				1.26 (0.76-2.08)				0.87 (0.49-1.54)
Serum creatinine >2 mg/dl				1.23 (0.37-4.08)				1.83 (0.53-6.32)

FEV_1_/Ht^3^: Forced expiratory volume in one second divided by height cubed; CVD: cardiovascular disease; COPD: chronic obstructive pulmonary diseases; hs-CRP: high sensitivity C-Reactive Protein; Model 1: adjusted for age and sex; Model 2: Model 1+ smoking status and other co-morbidities reported by GP; Model 3: Model 2+ anemia, hs-CRP and serum creatinine; *p-value < 0.05 **p-value < 0.01.

## Discussion

### Main findings and comparison with previous research

In a population-based prospective cohort of very old adults, we found that low FEV_1_ expressed as FEV_1_/Ht^3^ was associated with all-cause mortality, unplanned hospitalization as well as decline in mental and physical functioning independent of multiple potential confounders including age, smoking, chronic lung disease and an inflammation marker.

In previous clinical population studies including older adults, FEV_1_/Ht^3^ has been found to be better than FEV_1_% for predicting all-cause mortality, but it has not yet been investigated in relation to other adverse health outcomes [23,25,26]. Our study found the lowest quartile of FEV_1_/Ht^3^ to be associated with all-cause mortality in 3 years follow up of a cohort of very old adults who have not been the primary focus of previous studies. This association remained even after adjustment for a variety of potential confounders such as respiratory and non-respiratory co-morbidities, high levels of hs-CRP and serum creatinine that were not used in previous studies. In addition, our study explored the association of FEV_1_/Ht^3^ with other adverse outcomes such as unplanned hospitalizations and decline in physical and mental functioning that are particularly relevant for the very old adults.

In our cohort, those in the lowest FEV_1_/Ht^3^ quartile had a significantly higher risk for unplanned hospitalization during the 3 year follow-up, even after adjustment for multiple potential confounders. We also found that being in the lowest quartile of FEV_1_/Ht^3^ more than doubled the risk for mental decline (as defined for the purposes of our analysis) at 1.7 ± 0.21 years follow-up. Previous studies have found lower FEV_1_ (including different height- adjustments) to be associated with lower scores in cognitive tests and higher risk for cognitive decline over long term [7,12,13,15-17]. Our study investigated and confirmed for the first time the predictive value of FEV_1_/Ht^3^ for mental decline in a cohort of very old adults using standardized assessment of both cognitive and mood components.

Regarding physical decline, in line with previous studies where lower PF has been found to be associated with poor physical functioning [7-10,42], we found a significant association at baseline between FEV_1_/Ht^3^ and each of the physical functioning components used in our study (ADL score, PPT score and grip strength). Our study explored for the first time the association of FEV_1_ expressed as FEV_1_/Ht^3^ and decline in physical functioning over 1.7 ± 0.21 years follow-up in a cohort of very old adults using both self-reporting (ADL) and performance-based tests (PPT and grip strength). We found that the lowest quartile of FEV_1_/Ht^3^ had a statistically significant higher risk for physical decline only after adjustment for multiple confounders. These findings show a trend for a positive association of lowered FEV_1_/Ht^3^ with physical decline. Further investigations are needed to explore this association using longer follow-up time as well as other definitions and cut-offs for physical decline and its components.

Previous studies on the association of spirometry parameters of PF with adverse health in the older adults have used different standardizations of FEV_1_ and measures of physical and mental functioning and have also not focused on the growing population of very old adults. More longitudinal studies are needed in this area, especially as the association of spirometry-based respiratory impairment with frailty as well as their combined effect on mortality has been recently hypothesized and tested in adults 65–80 years old supporting the exploration of PF parameters as prognostic markers of frailty and adverse health outcomes in the older adults [18]. This is of interest as frailty is prevalent in the older adults and a precursor of disability and other adverse outcomes, but may be reversible in its early stages [43-45]. Yet, there is no consensus on the best instrument for assessment of frailty and in light of its multi-domain phenotype current focus is on cognitive, mood and social components beside the classical physical ones [46]. Our findings support the need for further research on the use of FEV_1_ as a predictor for important adverse outcomes and potential frailty marker in the very old adults and consideration as an indicator of overall health in geriatric assessments [1,7]. FEV_1_ standardized as FEV_1_/Ht^3^ takes into account the variability of body size and does not require the use of reference values and equations so it may be more suitable for use in this age group.

### Strengths and limitations

This study has several strengths. It is based on a large heterogeneous population representative of the very old adults in Belgium [28]. The protocol of the BF_c80+_ study included a comprehensive geriatric assessment that allowed for a rich analysis of different outcomes and risk factors. The same standardized examination and questionnaires were applied to all participants as the involved GPs and CRAs received training in order to standardize the data collection and recording. Mental and physical functioning was assessed with standardized and validated self-reported and performance-based tests. Rigorous quality control of spirometry performance and interpretation based on the ATS/ERS quality criteria were followed and various confounders were included in the analysis covering demographics, smoking status, non-respiratory and respiratory co-morbidities as well as a marker of systemic inflammation (hs-CRP).

The exclusion criteria in the selection of participants for the BF_c80+_ study (dementia or severe cognitive impairment and being in palliative or emergency care) are one of the limitations of this study. The definitions of physical and mental decline in our study were not based on a validated scoring method and the cut-off values for decline in some of the components may have missed or overestimated some of the change. We also used the actual instead of true height in our study. This becomes an issue in the older adults as height reduction is frequent in this age group due to both ageing and disease-related osteoporotic vertebral changes and introduces bias in lung function testing with possible overestimation of its values [26]. While the effect of height reduction has been found to be smallest on FEV_1_ and FVC, other proxies of height could be considered such as recalled tallest height or height calculated on arm span [47]. We plan to explore this in future studies as arm span has been measured in the BF_c80+_ study population.

## Conclusions

In a representative sample of adults 80 years and older, a low FEV_1_/Ht^3^ was an independent short-term predictor of all-cause mortality and hospitalizations as well as decline in physical and mental functioning. These findings support the consideration of FEV_1_/Ht^3^ as an alternative way of standardizing FEV_1_ and further exploration of its role as a potential risk marker for frailty and adverse health outcomes in very old adults.

## References

[1] Lange P (2011). Spirometric findings as predictors of survival. Thorax.

[2] Burney PG, Hooper R (2011). Forced vital capacity, airway obstruction and survival in a general population sample from the USA. Thorax.

[3] Schunemann HJ, Dorn J, Grant BJ, Winkelstein W, Trevisan M (2000). Pulmonary function is a long-term predictor of mortality in the general population: 29-year follow-up of the Buffalo Health Study. Chest.

[4] Sin DD, Wu L, Man SF (2005). The relationship between reduced lung function and cardiovascular mortality: a population-based study and a systematic review of the literature. Chest.

[5] Hole DJ, Watt GC, Davey-Smith G, Hart CL, Gillis CR, Hawthorne VM (1996). Impaired lung function and mortality risk in men and women: findings from the Renfrew and Paisley prospective population study. BMJ.

[6] Sabia S, Shipley M, Elbaz A, Marmot M, Kivimaki M, Kauffmann F (2010). Why does lung function predict mortality? results from the Whitehall II Cohort Study. Am J Epidemiol.

[7] Singh-Manoux A, Dugravot A, Kauffmann F, Elbaz A, Ankri J, Nabi H (2011). Association of lung function with physical, mental and cognitive function in early old age. Age (Dordrecht, Netherlands).

[8] Simpson CF, Punjabi NM, Wolfenden L, Shardell M, Shade DM, Fried LP (2005). Relationship between lung function and physical performance in disabled older women. J Gerontol A Biol Sci Med Sci.

[9] Thorpe RJ, Szanton SL, Whitfield K (2009). Association between lung function and disability in African-Americans. J Epidemiol Community Health.

[10] Fragoso CA, Gahbauer EA, Van Ness PH, Concato J, Gill TM (2008). Peak expiratory flow as a predictor of subsequent disability and death in community-living older persons. J Am Geriatr Soc.

[11] Cook NR, Albert MS, Berkman LF, Blazer D, Taylor JO, Hennekens CH (1995). Interrelationships of peak expiratory flow rate with physical and cognitive function in the elderly: MacArthur foundation studies of aging. J Gerontol A Biol Sci Med Sci.

[12] Chyou PH, White LR, Yano K, Sharp DS, Burchfiel CM, Chen R (1996). Pulmonary function measures as predictors and correlates of cognitive functioning in later life. Am J Epidemiol.

[13] Anstey KJ, Windsor TD, Jorm AF, Christensen H, Rodgers B (2004). Association of pulmonary function with cognitive performance in early, middle and late adulthood. Gerontology.

[14] Richards M, Strachan D, Hardy R, Kuh D, Wadsworth M (2005). Lung function and cognitive ability in a longitudinal birth cohort study. Psychosom Med.

[15] Guo X, Waern M, Sjogren K, Lissner L, Bengtsson C, Bjorkelund C (2007). Midlife respiratory function and Incidence of Alzheimer's disease: a 29-year longitudinal study in women. Neurobiol Aging.

[16] Vidal JS, Aspelund T, Jonsdottir MK, Jonsson PV, Harris TB, Lopez OL (2013). Pulmonary function impairment may be an early risk factor for late-life cognitive impairment. J Am Geriatr Soc.

[17] Weuve J, Glymour MM, Hu H, Sparrow D, Spiro A, Vokonas PS (2011). Forced expiratory volume in 1 second and cognitive aging in men. J Am Geriatr Soc.

[18] Vaz Fragoso CA, Enright PL, McAvay G, Van Ness PH, Gill TM (2012). Frailty and respiratory impairment in older persons. Am J Med.

[19] Vaz Fragoso CA, Gill TM, McAvay G, Quanjer PH, Van Ness PH, Concato J (2013). Respiratory impairment in older persons: when less means more. Am J Med.

[20] Stanojevic S, Wade A, Stocks J (2010). Reference values for lung function: past, present and future. Eur Respir J.

[21] Quanjer PH, Stanojevic S, Cole TJ, Baur X, Hall GL, Culver BH (2012). Multi-ethnic reference values for spirometry for the 3-95-yr age range: the global lung function 2012 equations. Eur Respir J.

[22] Loth DW, Ittermann T, Lahousse L, Hofman A, Leufkens HG, Brusselle GG (2013). Normal spirometry values in healthy elderly: the Rotterdam Study. Eur J Epidemiol.

[23] Chinn S, Gislason T, Aspelund T, Gudnason V (2007). Optimum expression of adult lung function based on all-cause mortality: results from the Reykjavik study. Respir Med.

[24] Miller MR, Pedersen OF, Lange P, Vestbo J (2009). Improved survival prediction from lung function data in a large population sample. Respir Med.

[25] Miller MR, Pedersen OF (2010). New concepts for expressing forced expiratory volume in 1 s arising from survival analysis. Eur Respir J.

[26] Pedone C, Scarlata S, Scichilone N, Forastiere F, Bellia V, Antonelli-Incalzi R (2013). Alternative ways of expressing FEV1 and mortality in elderly people with and without COPD. Eur Respir J.

[27] Pedone C, Scarlata S, Zito A, Forastiere F, Scichilone N, Battaglia S (2013). Alternative ways of expressing forced expiratory volume in the first second and long-term mortality in elderly patients with asthma. Ann Allergy Asthma Immunol.

[28] Vaes B, Pasquet A, Wallemacq P, Rezzoug N, Mekouar H, Olivier PA (2010). The BELFRAIL (BFC80+) study: a population-based prospective cohort study of the very elderly in Belgium. BMC Geriatr.

[29] Degryse J, Buffels J, Van Dijck Y, Decramer M, Nemery B (2012). Accuracy of office spirometry performed by trained primary-care physicians using the MIR Spirobank hand-held spirometer. Respiration.

[30] Miller MR, Hankinson J, Brusasco V, Burgos F, Casaburi R, Coates A (2005). Standardisation of spirometry. Eur Respir J.

[31] McWhinnie JR (1981). Disability assessment in population surveys: results of the O.E.C.D. Common Development Effort. Rev Epidemiol Sante Publique.

[32] Speer DC, Greenbaum PE (1995). Five methods for computing significant individual client change and improvement rates: support for an individual growth curve approach. J Consult Clin Psychol.

[33] Guralnik JM, Simonsick EM, Ferrucci L, Glynn RJ, Berkman LF, Blazer DG (1994). A short physical performance battery assessing lower extremity function: association with self-reported disability and prediction of mortality and nursing home admission. J Gerontol.

[34] Guralnik JM, Ferrucci L, Simonsick EM, Salive ME, Wallace RB (1995). Lower-extremity function in persons over the age of 70 years as a predictor of subsequent disability. N Engl J Med.

[35] Gawel J, Vengrow D, Collins J, Brown S, Buchanan A, Cook C (2012). The short physical performance battery as a predictor for long term disability or institutionalization in the community dwelling population aged 65 years old or older. Phys Ther Rev.

[36] Giampaoli S, Ferrucci L, Cecchi F, Lo Noce C, Poce A, Dima F (1999). Hand-grip strength predicts incident disability in non-disabled older men. Age Ageing.

[37] Taekema DG, Gussekloo J, Maier AB, Westendorp RG, de Craen AJ (2010). Handgrip strength as a predictor of functional, psychological and social health. A prospective population-based study among the oldest old. Age Ageing.

[38] Tombaugh TN, McIntyre NJ (1992). The mini-mental state examination: a comprehensive review. J Am Geriatr Soc.

[39] Sheikh JI YJ: Geriatric Depression Scale (GDS): Recent evidence and development of a shorter version. In Clinical Gerontology: A Guide to Assessment and Intervention. Edited by TL B. NY: Haworth Press; 1986: 165–173

[40] de Craen AJ, Heeren TJ, Gussekloo J (2003). Accuracy of the 15-item geriatric depression scale (GDS-15) in a community sample of the oldest old. Int J Geriatr Psychiatry.

[41] Marc LG, Raue PJ, Bruce ML (2008). Screening performance of the 15-item geriatric depression scale in a diverse elderly home care population. Am J Geriatr Psychiatry.

[42] Eisner MD, Iribarren C, Yelin EH, Sidney S, Katz PP, Ackerson L (2008). Pulmonary function and the risk of functional limitation in chronic obstructive pulmonary disease. Am J Epidemiol.

[43] Santos-Eggimann B, Cuenoud P, Spagnoli J, Junod J (2009). Prevalence of frailty in middle-aged and older community-dwelling Europeans living in 10 countries. J Gerontol A Biol Sci Med Sci.

[44] Fried LP, Tangen CM, Walston J, Newman AB, Hirsch C, Gottdiener J (2001). Frailty in older adults: evidence for a phenotype. J Gerontol A Biol Sci Med Sci.

[45] Gill TM, Gahbauer EA, Allore HG, Han L (2006). Transitions between frailty states among community-living older persons. Arch Intern Med.

[46] Sternberg SA, Schwartz AW, Karunananthan S, Bergman H, Mark Clarfield A (2011). The identification of frailty: a systematic literature review. J Am Geriatr Soc.

[47] Kjensli A, Ryg M, Falch JA, Armbrecht G, Diep LM, Eriksen EF (2010). Does body height reduction influence interpretation of lung function in COPD patients?. Eur Respir J.

